# Supplementary material for: GhostFold: Accurate protein structure prediction using structure-constrained synthetic coevolutionary signals
Source: bioRxiv. 2025 Oct 14:2025.10.13.682177. Preprint. [Version 1] doi: 10.1101/2025.10.13.682177 (PMC12633075; doi:10.1101/2025.10.13.682177)
Supplement: Supplement 1 [file NIHPP2025.10.13.682177v1-supplement-1.pdf]

bioRxiv preprint doi: https://doi.org/10.1101/2025.10.13.682177; this version posted October 14, 2025. The copyright holder for this preprint (which was not certified by peer review) is the author/funder, who has granted bioRxiv a license to display the preprint in perpetuity. It is made available under aCC-BY 4.0 International license.

SUPPLEMENTARY INFORMATION

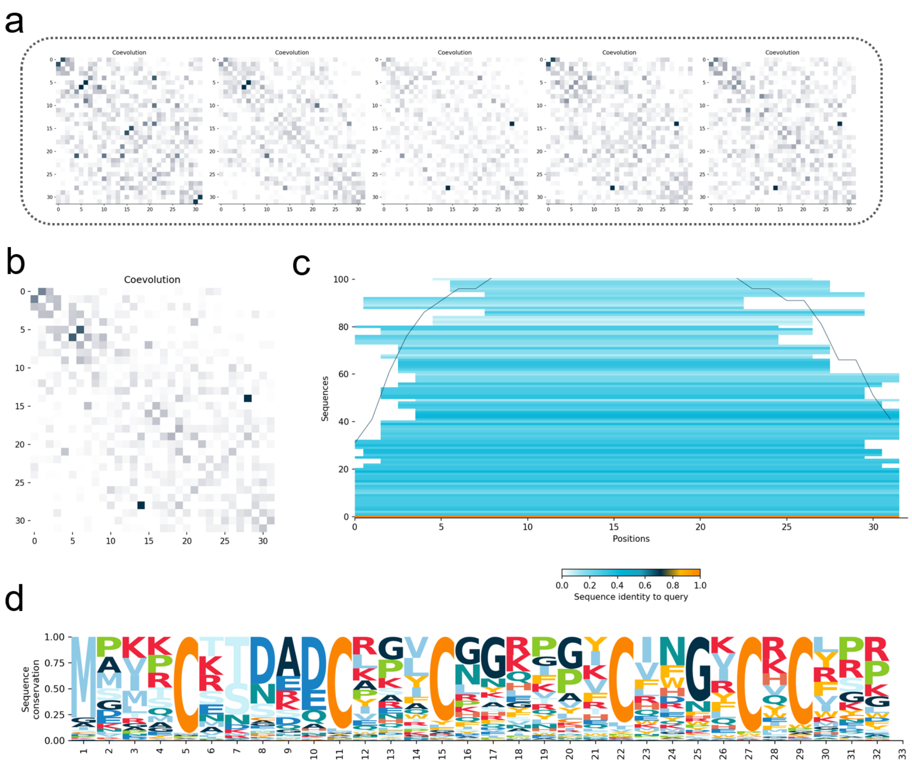

Supplementary Figure 1 - Emergence of coevolutionary signal and structural coherence in synthetically generated pseudoMSAs. (a) Pairwise contact probability matrices from representative back-translated sequences generated using the AA→3Di→AA pipeline, visualized as individual covariation heatmaps. Each map is sparse and noisy in isolation, capturing only weak coevolutionary signals due to the limited sequence length and stochastic nature of individual samples. (b) Aggregated coevolution matrix derived from the full pseudoMSA, constructed from 100 synthetically generated sequences. Despite originating from de novo generation, the alignment exhibits robust long-range coevolutionary couplings between distal residues—hallmarks of structural constraints encoded within the β-grasp fold of the input query (6MRQ). (c) Positional coverage and diversity of the generated pseudoMSA. Horizontal bars represent individual sequences, colored by sequence identity to the original query. Sequences span from full-length to partial fragments (coverage 40–100%) to enhance local structural motif diversity. The grey line indicates total coverage per position, showing uniform representation across the full sequence. (d) Sequence logo representing positional amino acid preferences across the pseudoMSA. The alignment captures conserved structural features of the β-grasp fold, including glycine and proline residues at key positions and terminal cysteines involved in disulfide bridge formation. The variability in other positions reflects controlled evolutionary drift induced via stochastic decoding and substitution-matrix-guided mutagenesis.

bioRxiv preprint doi: https://doi.org/10.1101/2025.10.13.682177; this version posted October 14, 2025. The copyright holder for this preprint (which was not certified by peer review) is the author/funder, who has granted bioRxiv a license to display the preprint in perpetuity. It is made available under aCC-BY 4.0 International license.

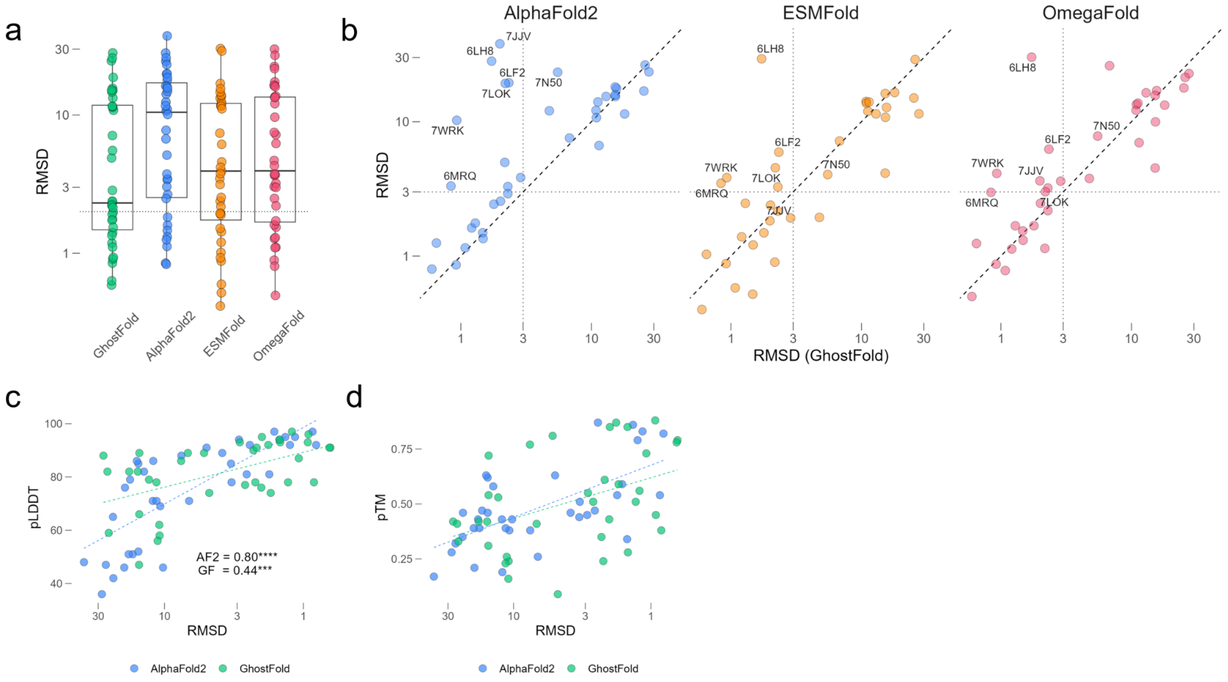

Supplementary Figure 2. GhostFold yields consistently lower RMSD and alters AlphaFold2 confidence score interpretation. (a) Distribution of RMSD values for all 36 orphan protein targets across GhostFold, AlphaFold2, ESMFold, and OmegaFold. Boxplots show median, interquartile range, and individual data points (log scale). GhostFold displays a tighter and lower RMSD distribution, indicative of overall higher accuracy. (b) Extended RMSD comparison plots for GhostFold versus AlphaFold2, ESMFold, and OmegaFold, including all 36 orphan proteins. This includes the 12 “failure” cases where all predictors had RMSD > 3 Å. (c) Correlation between RMSD and per-residue confidence scores (pLDDT) for AlphaFold2 (blue) and GhostFold (green). While AF2 shows a strong negative correlation ($R^2 = 0.80$), GhostFold’s correlation, though significant, is noticeably weaker ($R^2 = 0.44$), indicating a potential shift in the interpretability of pLDDT scores when using synthetic pseudoMSAs. (d) Correlation between RMSD and predicted TM-score (pTM) for AlphaFold2 and GhostFold. GhostFold models tend to have lower pTM values overall (< 0.5), but a weak linear trend remains. The divergence in confidence metrics underscores the need for alternative confidence calibration when using pseudoMSAs.

bioRxiv preprint doi: https://doi.org/10.1101/2025.10.13.682177; this version posted October 14, 2025. The copyright holder for this preprint (which was not certified by peer review) is the author/funder, who has granted bioRxiv a license to display the preprint in perpetuity. It is made available under aCC-BY 4.0 International license.

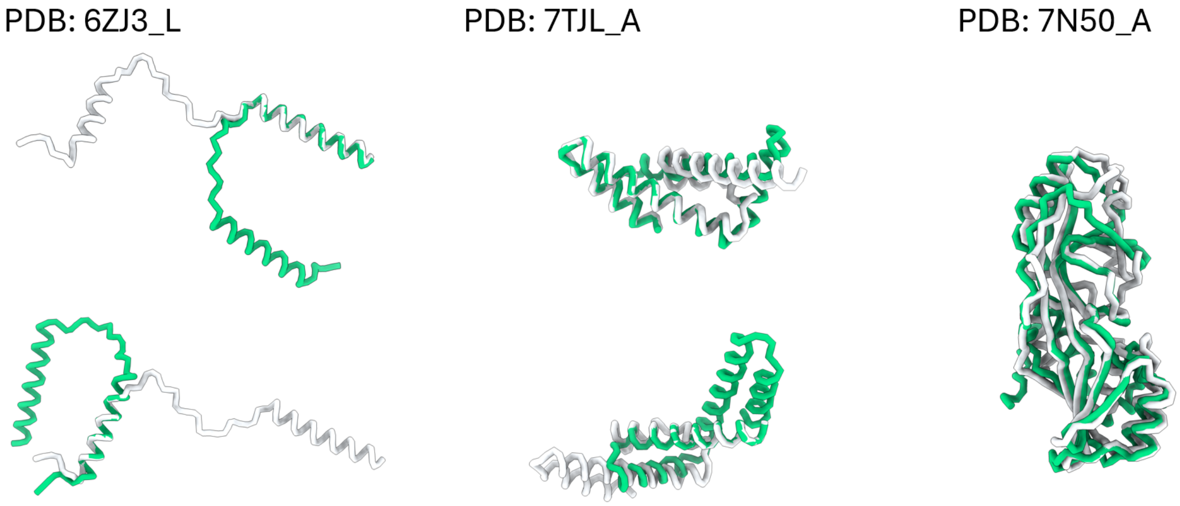

Supplementary Figure 3. GhostFold recovers correct domains in globally "failed" targets. For 12 of 36 orphan proteins, no method produced a fully correct full-length model; nevertheless, GhostFold recapitulates most constituent domains in several of these failures. Shown are three representative cases (6ZJ3_L, 7TJL_A, 7N50_A). For each protein, shown are the global superposition of the native structure (gray) and the GhostFold prediction (green), illustrating incorrect inter-domain packing/relative orientation that inflates the full-chain RMSD.

bioRxiv preprint doi: https://doi.org/10.1101/2025.10.13.682177; this version posted October 14, 2025. The copyright holder for this preprint (which was not certified by peer review) is the author/funder, who has granted bioRxiv a license to display the preprint in perpetuity. It is made available under aCC-BY 4.0 International license.

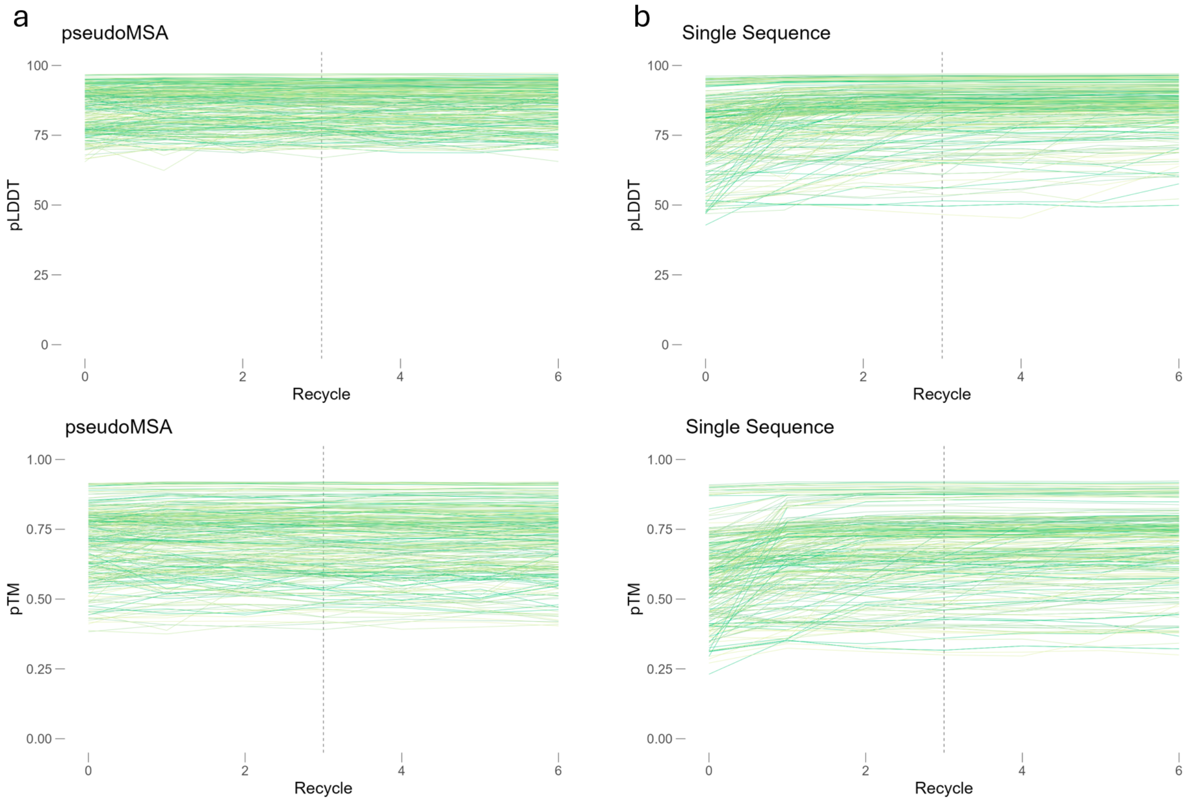

Supplementary Figure 4. GhostFold yields consistently high confidence scores across all AlphaFold2 model variants. (a–b) Line plots showing pLDDT (top) and pTM (bottom) values across recycling steps (x-axis) for all five AlphaFold2 model variants (different shades of green) on the Design55 dataset. Each line represents one design prediction from a given model. (a) GhostFold (pseudoMSA input) maintains consistently high per-residue (pLDDT) and global fold (pTM) confidence across all five AlphaFold2 models. Confidence scores remain stable from recycle 0 onward, indicating minimal dependence on iterative refinement. (b) In contrast, the standard single-sequence mode (without pseudoMSA) exhibits greater variance and a broader dynamic range in both pLDDT and pTM values. Confidence scores generally increase with recycling, indicating that the model relies on iterative refinement to achieve convergence in the absence of strong input signal. Vertical dashed lines indicate the transition from early to later recycling iterations (recycle 3). Across all five model variants, GhostFold provides structurally informative pseudoMSAs that result in confident predictions from the very first recycle, underscoring the robustness of the approach across AlphaFold2's model ensemble.

bioRxiv preprint doi: https://doi.org/10.1101/2025.10.13.682177; this version posted October 14, 2025. The copyright holder for this preprint (which was not certified by peer review) is the author/funder, who has granted bioRxiv a license to display the preprint in perpetuity. It is made available under aCC-BY 4.0 International license.

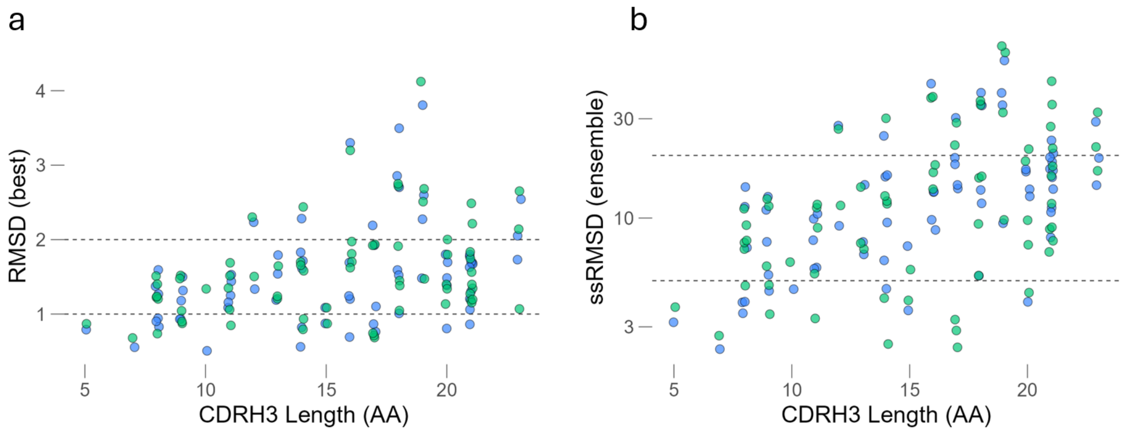

Supplementary Figure 5. GhostFold ensemble consistency is robust to loop length and model variance. (a) Per-target best CDRH3 RMSD as a function of loop length. GhostFold (green) maintains low RMSD across increasing CDRH3 lengths, while AlphaFold2 (blue) shows degraded performance for loops longer than 15 residues. Dashed lines at 1 Å and 2 Å indicate accuracy benchmarks for loop modeling. (b) Ensemble ssRMSD plotted against CDRH3 loop length. GhostFold remains stable and compact across all lengths, while AlphaFold2 shows increasing variability with longer loops. Together with panel B, this highlights GhostFold's unique ability to maintain both accuracy and consistency across difficult hypervariable regions.
